# Role of spinal P2Y_6_ and P2Y_11_ receptors in neuropathic pain in rats: possible involvement of glial cells

**DOI:** 10.1186/1744-8069-10-29

**Published:** 2014-05-20

**Authors:** Paulino Barragán-Iglesias, Jorge Baruch Pineda-Farias, Claudia Cervantes-Durán, Mariana Bravo-Hernández, Héctor Isaac Rocha-González, Janet Murbartián, Vinicio Granados-Soto

**Affiliations:** 1 Neurobiology of Pain Laboratory, Departamento de Farmacobiología, Centro de Investigación y de Estudios Avanzados (Cinvestav), Sede Sur, Calzada de los Tenorios 235, Colonia Granjas Coapa, 14330 México, D.F., México; 2 Sección de Estudios de Posgrado e Investigación, Escuela Superior de Medicina, Instituto Politécnico Nacional, México D.F., México

**Keywords:** Astrocytes, Microglia, Neuropathic pain, P2Y_6_ receptors, P2Y_11_ receptors, Spinal cord

## Abstract

**Background:**

The participation of spinal P2X receptors in neuropathic pain is well recognized. However, the role of P2Y receptors has been less studied. The purpose of this study was to investigate the contribution of spinal P2Y_6,11_ receptors following peripheral nerve damage induced by spinal nerve ligation. In addition, we determined the expression of P2Y_6,11_ receptors in the dorsal spinal cord in presence of the selective P2Y_6,11_ receptors antagonists. Furthermore, we evaluated the participation of spinal microglia and astrocytes in the pronociceptive role of P2Y_6,11_ receptors.

**Results:**

Spinal administration of the selective P2Y_6_ (MRS2578, 10–100 μM) and P2Y_11_ (NF340, 0.3–30 μM) receptor antagonists reduced tactile allodynia in spinal nerve ligated rats. Nerve injury increased the expression of P2Y_6,11_ receptors at 7, 14 and 21 days after injury. Furthermore, intrathecal administration of MRS2578 (100 μM/day) and NF340 (30 μM/day) for 3 days significantly reduced spinal nerve injury-induced increase in P2Y_6_,_11_ receptors expression, respectively. Spinal treatment (on day 14 after injury) with minocycline (100 μg/day) or fluorocitrate (1 nmol/day) for 7 days reduced tactile allodynia and spinal nerve injury-induced up-regulation in Iba-1 and GFAP, respectively. In addition, minocycline reduced nerve injury-induced up-regulation in P2Y_6,11_ receptors whereas that fluorocitrate diminished P2Y_11_, but not P2Y_6_, receptors up-regulation. Intrathecal treatment (on day 21 after injury) with the selective P2Y_6_ (PSB0474, 3–30 μM) and P2Y_11_ (NF546, 1–10 μM) receptor agonists produced remarkable tactile allodynia in nerve ligated rats previously treated with minocycline or fluorocitrate for 7 days.

**Conclusions:**

Our data suggest that spinal P2Y_6_ is present in spinal microglia while P2Y_11_ receptors are present in both spinal microglia and astrocytes, and both receptors are up-regulated in rats subjected to spinal nerve injury. In addition, our data suggest that the spinal P2Y_6_ and P2Y_11_ receptors participate in the maintenance of neuropathic pain.

## Background

ATP (adenosine triphosphate) is released from primary afferents following peripheral tissue/nerve damage producing pain [1,2]. The pronociceptive role of ATP in nociception is well characterized [3-8]. This effect has been associated with the activation of the P2X family receptors expressed in dorsal root ganglion [9-12], satellite glial cells [13,14], spinal dorsal horn [15-17] and microglia/astrocytes [16,18,19]. Following nerve damage, spinal microglia and astrocytes proliferate and up-regulate specific protein markers like ionized calcium-binding adapter molecule-1 (Iba-1) and glial fibrillary acidic protein (GFAP), respectively [20,21]. It is thought that these changes are involved in neuropathic pain as activated microglia and astrocytes have the ability to modify and modulate neuronal firing and to release pro- and anti-inflammatory molecules. Importantly, spinal microglia is activated by ATP through P2X_4_ and P2X_7_ receptors leading to release of pro-inflammatory cytokines (interleukin-1β [IL-1β]), tumor necrosis factor α [TNFα], interleukin-6 [IL-6]), chemokines (CCL-2, fractalkine) and neurotrophic factors (brain derived neurotrophic factor [BDNF]), among others, to produce central sensitization [22-24].

In comparison to P2X receptors, the contribution of P2Y receptors to neuropathic pain has been less studied. At the moment, there is limited evidence to suggest that P2Y receptors play a role in neuropathic pain. However, data have shown that P2Y_1_ receptors mRNA is up-regulated in rat dorsal root ganglion neurons after peripheral axotomy of the sciatic nerve [25]. Likewise, P2Y_1_ and P2Y_2_ receptors mRNAs are up-regulated in mouse L2/L3 dorsal root ganglion neurons after nerve transection [26]. P2Y receptors might also be involved in spinal microglia activation during neuropathic pain conditions. In this sense, P2Y_12_ receptors are up-regulated in spinal microglia after peripheral nerve injury [27]. Moreover, P2Y_6_, P2Y_13_ and P2Y_14_ mRNAs are up-regulated in microglia in the ipsilateral spinal cord following nerve injury [28]. Furthermore, recent evidence suggests that some P2Y receptors are expressed in satellite glial cells and astrocytes and they contribute to neuropathic pain following nerve damage [29].

Due to the limited information about the participation of P2Y receptors in neuropathic pain, we decided to study the role of spinal P2Y_6_ and P2Y_11_ receptors in the maintenance of neuropathic pain in the rat by pharmacological and molecular methods. Our data suggest that spinal P2Y_6_ is present in spinal microglia while P2Y_11_ receptors are present in both spinal microglia and astrocytes, and both receptors are up-regulated in rats subjected to spinal nerve injury. In addition, our data suggest that the spinal P2Y_6_ and P2Y_11_ receptors participate in the maintenance of neuropathic pain.

## Results

### Antiallodynic effects of selective P2Y_6_ (MRS2578) and P2Y_11_ (NF340) receptor antagonists in rats

**Figure 1:**
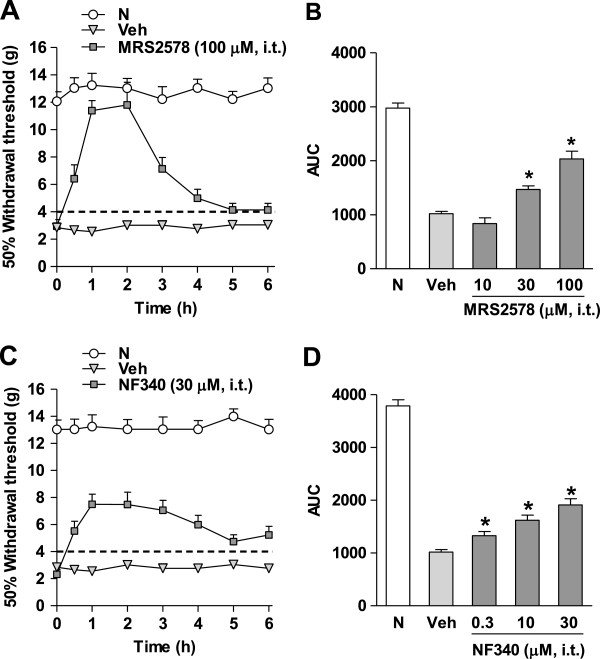
**Intrathecal injection of the selective P2Y**_**6,11**_**receptor antagonists reverses tactile allodynia.** Time course of the antiallodynic effects of the selective P2Y_6_ (MRS2578, panel **A**) and P2Y_11_ (NF340, panel **C**) receptor antagonists in rats submitted to spinal nerve injury. Data are expressed as mean ± S.E.M. for 6 animals. Withdrawal threshold was assessed 14 days after spinal nerve injury. Dose–response curves of the antiallodynic effect of MRS2578 (10–100 μM, panel **B**) and NF340 (0.3–30 μM, panel **D**). Data are expressed as the 50% threshold withdrawal against time curve (AUC). * Significantly different from the vehicle (Veh) group (*p* < 0.05), as determined by analysis of variance followed by the Student-Newman-Keuls test. N: Naive.

Spinal nerve injury produced tactile allodynia in the ipsilateral hind paw, which was evident by a significant decrease in the 50% paw withdrawal threshold response as compared to the naive group (Figure 1A/C). As expected, sham surgery did not change the 50% withdrawal threshold response on the ipsilateral side as previously reported by our group [30]. Furthermore, intrathecal administration of vehicle did not affect tactile allodynia induced by spinal nerve ligation (Figure 1A/C). In marked contrast, the spinal administration of the selective P2Y_6_ (MRS2578, Figure 1A) and P2Y_11_ (NF340, Figure 1C) receptor antagonists produced an antiallodynic effect in the spinal nerve injured rats. The maximal antiallodynic effect in both cases was reached in about 1 h and then decayed gradually in about 6 h. However, MRS2578 had a greater peak effect than NF340, although the effect of the later decayed more slowly. MRS2578 (10–100 μM, Figure 1B) and NF340 (0.3–30 μM, Figure 1D) reversed significantly (*p* < 0.05) and dose-dependently tactile allodynia in the spinal nerve injured rats.

### Expression of P2Y_6_ and P2Y_11_ receptors in spinal nerve injured rats: effects of MRS2578 and NF340

**Figure 2:**
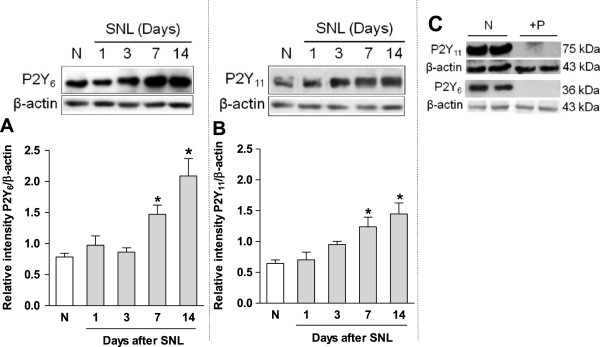
**Spinal nerve injury enhances the expression of P2Y**_**6,11**_**receptors.** Western blot analysis of the P2Y_6_ (panel **A**) and P2Y_11_ (panel **B**) receptors expression in the ipsilateral dorsal spinal cord obtained from naïve and spinal nerve injured (SNL) rats. The primary antibody for P2Y_6_ and P2Y_11_ were pre-adsorbed with the corresponding antigenic peptide (+P, panel **C**). Data were normalized against β-actin and are expressed as the mean ± S.E.M. of 3 independent rats. *Significantly (*p* < 0.05) different from the naïve (N) group, as determined by one-way analysis of variance followed by the Student-Newman-Keuls test.

Western blot analysis showed bands of about 36 and 75 kDa for P2Y_6_ (Figure 2A) and P2Y_11_ (Figure 2B) receptors at the ipsilateral dorsal spinal cord, respectively. Spinal nerve ligation produced a significant (*p* < 0.05) increase in the expression of P2Y_6_ and P2Y_11_ receptors at 7 and 14 days (Figure 2A/B), as well as 21 days after nerve injury (Additional file 1: Figure S1). No significant (*p* > 0.05) increase of P2Y_6_ (Additional file 2: Figure S2A) and P2Y_11_ (Additional file 2: Figure S2B) receptors expression was observed in the contralateral dorsal spinal cord. There were no immunoreactive bands for both receptors when the primary antibodies were pre-adsorbed with the corresponding antigenic peptides (Figure 2C).

**Figure 3:**
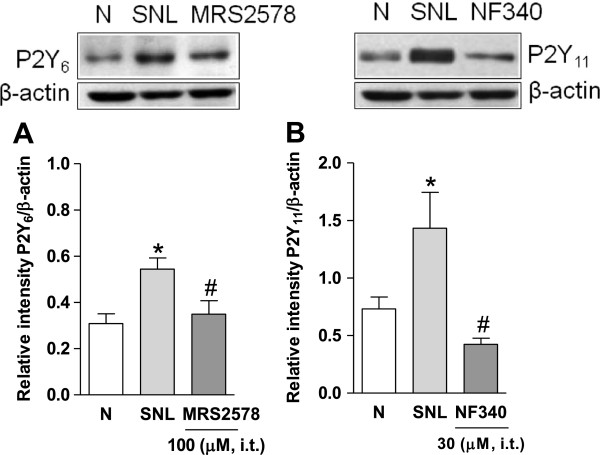
**P2Y**_**6,11**_**receptor antagonists prevent enhanced expression of P2Y**_**6,11**_**receptors.** Effect of the repeated intrathecal administration of MRS2578 (panel **A**) and NF340 (panel **B**) in spinal nerve injury-induced increase in P2Y_6_ and P2Y_11_ expression in the ipsilateral dorsal spinal cord. Data were normalized against β-actin and are expressed as the mean ± S.E.M. of 3 independent rats. * Significantly (*p* < 0.05) different from the naïve (N) group and ^#^ significantly (*p* < 0.05) different from the spinal nerve ligated (SNL) group, as determined by one-way analysis of variance followed by the Student-Newman-Keuls test.

Repeated intrathecal administration (starting on day 12 after nerve injury) of the selective P2Y_6_ (MRS2578, 100 μM/12 h for 3 days) and P2Y_11_ (NF340, 30 μM/12 h for 3 days) receptor antagonists, but not vehicle, significantly (*p* < 0.05) reversed spinal nerve ligation-induced increase in P2Y_6_ (Figure 3A) and P2Y_11_ (Figure 3B) receptors expression in the dorsal spinal cord on day 14 after nerve injury.

### Effect of minocycline or fluorocitrate on tactile allodynia, glial markers, and P2Y_6,11_ receptors expression

**Figure 4:**
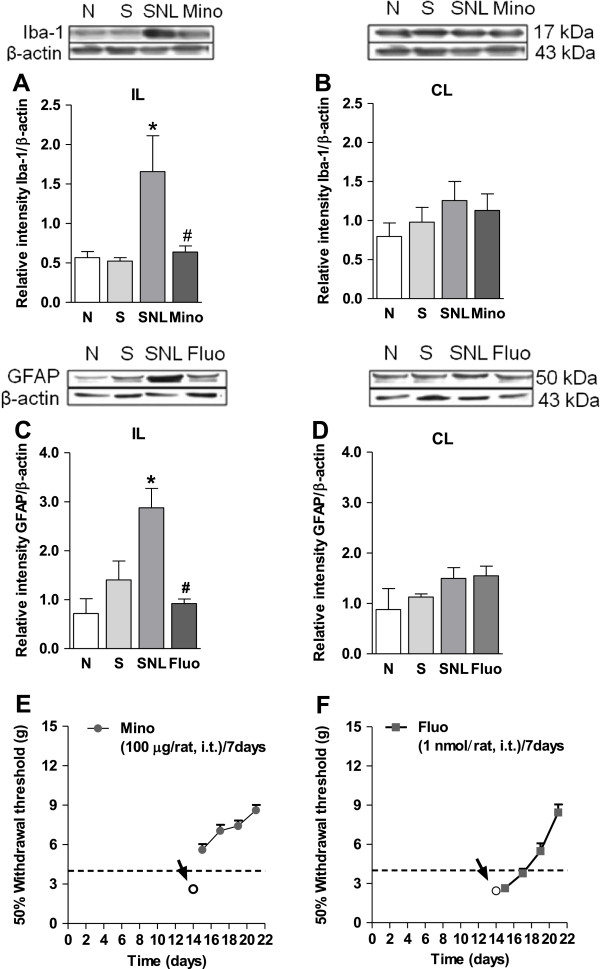
**Spinal nerve injury increases the expression of Iba-1 and GFAP: Blockade by minocycline or fluorocitrate.** Effect of the repeated intrathecal administration of minocycline (Mino, panel **A** and **B**) or fluorocitrate (Fluo, panel **C** and **D**) in spinal nerve injury-induced increased expression of Iba-1 and GFAP in the ipsilateral (IL) and contralateral (CL) dorsal spinal cord. Minocycline (panel **E**) and fluorocitrate (panel **F**) also reduced tactile allodynia in spinal nerve ligated rats. Data were normalized against β-actin and are expressed as the mean ± S.E.M. of 3 independent rats. * Significantly (*p* < 0.05) different from the sham (S) or naïve (N) groups and ^#^ significantly (*p* < 0.05) different from the spinal nerve ligated (SNL) group, as determined by one-way analysis of variance followed by the Student-Newman-Keuls test.

Spinal nerve injury produced tactile allodynia as well as a significant (*p* < 0.05) increase in the expression of microglia and astrocytes markers Iba-1 (Figure 4A) and GFAP (Figure 4C), respectively, 21 days later. This increase was only evident in the ipsilateral, but not contralateral, section of the dorsal spinal cord (Figure 4B/D).

**Figure 5:**
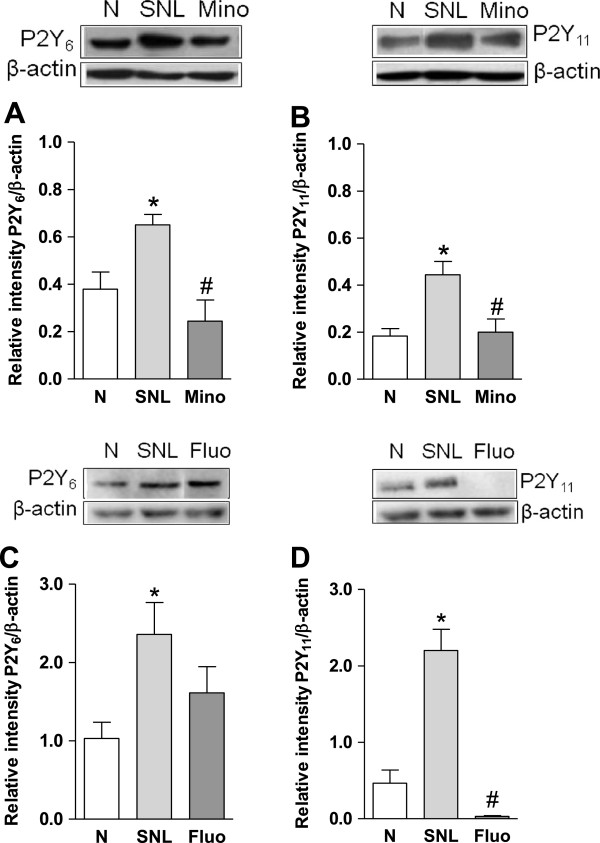
**Spinal nerve injury increases the expression of P2Y**_**6**_**and P2Y**_**11**_**: Blockade by minocycline or fluorocitrate.** Effect of the repeated intrathecal administration of minocycline (Mino, panel **A** and **B**) or fluorocitrate (Fluo, panel **C** and **D**) in spinal nerve injury-induced increased expression of P2Y_6_ and P2Y_11_ receptors in the ipsilateral dorsal spinal cord. Data were normalized against β-actin and are expressed as the mean ± S.E.M. of 3 independent rats. * Significantly (*p* < 0.05) different from the naïve (N) group and ^#^ significantly (*p* < 0.05) different from the spinal nerve ligated (SNL) group, as determined by one-way analysis of variance followed by the Student-Newman-Keuls test.

Once established tactile allodynia (14 days after nerve injury), repeated intrathecal treatment with minocycline (100 μg/day for 7 days, Figure 4E) or fluorocitrate (1 nmol/day for 7 days, Figure 4F), but not vehicle, reduced tactile allodynia in the spinal nerve ligated rats and diminished the increase in microglia and astrocytes markers Iba-1 (Figure 4A) and GFAP (Figure 4C) in the ipsilateral, but not contralateral, section of the dorsal spinal cord (Figure 4B/D). Moreover, repeated intrathecal administration of minocycline significantly (*p* < 0.05) reduced spinal nerve injury-induced increase in P2Y_6_ (Figure 5A) and P2Y_11_ (Figure 5B) expression in rats. In contrast, repeated intrathecal injection of fluorocitrate only diminished the increase of P2Y_11_ (Figure 5D), but not P2Y_6_ (Figure 5C), receptors expression in the same schedule.

### Effect of selective P2Y_6_ (PSB0474) and P2Y_11_ (NF546) receptor agonists in spinal nerve injured rats pre-treated with minocycline or fluorocitrate

Once established tactile allodynia (14 days), repeated intrathecal treatment with minocycline (100 μg/day for 7 days, Additional file 3: Figure S3A/B) or fluorocitrate (1 nmol/day for 7 days, Additional file 3: Figures S3C/D) reversed spinal nerve injury-induced tactile allodynia. On this condition, a bolus intrathecal injection with the selective P2Y_6_ (PSB0474, 3–30 μM; Additional file 3: Figure S3A/C) and P2Y_11_ (NF546, 1–10 μM; Additional file 3: Figure S3B/D) receptor agonists produced a remarkable and long-lasting tactile allodynia in the rats pretreated with minocycline or fluorocitrate. In contrast, intrathecal administration of vehicles did not modify the withdrawal threshold (Additional file 3: Figure S3A-D).

## Discussion

This study demonstrates that intrathecal administration of MRS2578 and NF340 partially reverses spinal nerve injury-induced tactile allodynia. Since these drugs are selective P2Y_6_[31] and P2Y_11_[32] receptor antagonists, respectively, our data suggest that both spinal receptors participate in the maintenance of neuropathic pain induced by spinal nerve injury. Our results agree with a previous report showing that intrathecal administration of MRS2578 suppresses spared nerve injury-induced mechanical allodynia [28], suggesting that spinal P2Y_6_ receptors participate in the maintenance of neuropathic pain. However, our data disagree with other study demonstrating that P2Y_6_ receptors play an antinociceptive role as intrathecal uridine diphosphate (UDP) administration (endogenous agonist) shows antiallodynic effects after partial sciatic nerve ligation [33]. This discrepancy could be explained on the basis of the used P2Y_6_ receptor agonist selectivity as UDP also activates the P2Y_14_ receptor with higher affinity than for P2Y_6_ receptors [34]. In our study we demonstrated that intrathecal administration of the selective P2Y_6_ (PSB0474) and P2Y_11_ (NF546) receptor agonists produces long-lasting tactile allodynia which argues against the antinociceptive effect of spinal P2Y_6_ receptors. Regarding NF340, this seems to be the first report demonstrating the antinociceptive effect of this compound suggesting that spinal P2Y_11_ receptors play an important role in the maintenance of neuropathic pain. The contribution of P2Y_6_ receptors seems to be different from that of P2Y_11_ receptors as the P2Y_6_ receptor antagonist produced a peak in the antiallodynic effect in about 1–2 h and then decayed rapidly. In contrast, the P2Y_11_ receptor antagonist produced a modest but sustained antiallodynic effect. This discrepancy could be due to differences in the pharmacokinetics or pharmacodynamics of the tested drugs. Taken together, our data suggest the participation of spinal P2Y_6,11_ receptors in the maintenance of neuropathic pain.

In support to our pharmacological study, we demonstrated by western blot that P2Y_6,11_ receptors are expressed in the dorsal spinal cord of naïve rats. Previous studies indicate that P2Y_6_ receptor mRNA is found in rat microglia [35]. On the contrary, P2Y_11_ receptors have only been described in humans. Currently there are no reports of a cloned P2Y_11_ receptor in rats. However, this receptor has been recently reported in epithelial cells [36], primary glial cells [37], lacrimal glands [38], neutrophils [39], and brain [38] of rats. Thus, our data suggest that the P2Y_6_ receptor is expressed and completely functional in the spinal cord. In addition, our results suggest the presence of a P2Y_11_–like receptor in the rat spinal cord. Besides their presence in naïve animals, P2Y_6,11_ receptors expression in the ipsilateral dorsal spinal cord was up-regulated at 7 and 14 days after spinal nerve ligation. These results partially agree with a previous study showing that P2Y_6_ receptor mRNA is up-regulated in the ipsilateral spinal cord at 3 days following peripheral nerve injury [28]. This time difference may be due to the neuropathic pain model or the method used to quantify the expression of the P2Y_6_ receptor (PCR *versus* WB). In the case of P2Y_11_ receptors, this is the first report about their increased expression. To further reinforce the participation of spinal P2Y_6,11_ receptors in the maintenance of neuropathic pain in rats, we demonstrated that intrathecal administration of the P2Y_6,11_ receptor antagonists reverses spinal nerve injury-induced tactile allodynia and P2Y_6,11_ receptors up-regulation. Admittedly, the mechanisms for the suppressive effects of P2Y_6,11_ receptor antagonists on the up-regulation of these receptors in the dorsal spinal cord after nerve injury are unclear. However, it is likely that blockade of both receptors may lead to a fall in central sensitization that then could promote the reduction in P2Y_6,11_ receptors expression. Clearly, further experiments which fall beyond the scope of our study will be required to confirm our suggestion.

It is accepted that spinal microglia and astrocytes release several pro-inflammatory mediators in the dorsal horn following nerve damage [22,24]. We confirmed the participation of microglia and astrocytes by showing that the ipsilateral, but not contralateral, spinal Iba-1 and GFAP are up-regulated in spinal nerve injured rats and this up-regulation is prevented by repeated administration of intrathecal minocycline or fluorocitrate, respectively. More important, spinal nerve injury increased expression of P2Y_6,11_ receptors in the dorsal spinal cord whereas that treatment with minocycline reduced spinal nerve damage-induced P2Y_6,11_ receptors enhanced expression. Thus, our data seem to point out that spinal P2Y_6,11_ receptors are present in activated microglia and they participate in the maintenance of neuropathic pain in the rat. In support of this, Kobayashi and coworkers reported the enhanced presence of P2Y_6_,_13_,_14_ receptors mRNA in spinal microglia following peripheral nerve injury [28]. Furthermore, intrathecal administration of the microglial p-38 MAPK inhibitor SB203580 suppressed spinal nerve injury-induced increase of P2Y_6_ mRNA suggesting that activated microglia leads to an enhanced expression of P2Y_6_ receptors and neuropathic pain [28].

We showed that fluorocitrate reduces spinal P2Y_11_, but not P2Y_6_, receptors up-regulation suggesting that activated astrocytes are over-expressing P2Y_11_ receptors and this phenomenon contributes to maintenance of neuropathic pain. In support of this, P2Y_11_ receptors have been found in astrocytes [37]. However, the fact that intrathecal administration of selective agonists of the P2Y_6,11_ receptors produced tactile allodynia once microglia and astrocytes have been previously blocked suggests that spinal P2Y_6,11_ receptors may participate promoting neuropathic pain at least partially in a microglia- or astrocytes-independent way. Supporting this, P2Y_6_, but not P2Y_11_, receptors mRNA have been found in dorsal root ganglion [26,40,41]. Taken together, data suggest that P2Y_6,11_ receptors are present in microglia while P2Y_11_ receptors are present only in astrocytes.

We found that repeated injections (for 7 days) of minocycline or fluorocitrate produced an antiallodynic effect in neuropathic rats. These data suggest that microglia and astrocytes play an important role in the maintenance phase of neuropathic pain. These data agree with previous observations showing that repeated treatment with minocycline reverses thermal hyperalgesia and mechanical allodynia [42-46]. In contrast, others have reported that minocycline prevents but do not reverses established neuropathic pain [47-49]. Differences could be due to the different models of neuropathic pain used. Regarding fluorocitrate, this drug reverses both ipsilateral and mirror-image spinal nerve injury-induced allodynia [49,50] which agree with our observations.

## Conclusion

Our data suggest that spinal P2Y_6_,_11_ receptors are present in spinal microglia while P2Y_11_ receptors are present only in astrocytes. Both receptors are up-regulated in rats subjected to spinal nerve injury. Our data suggest that the spinal P2Y_6_ and P2Y_11_ receptors participate in the maintenance of neuropathic pain.

## Methods

### Animals

Since previous experiments in our conditions found no differences between male and female rats [30], all experiments were performed on female Wistar rats (140–180 g). Animals were obtained from our own breeding facilities and had free access to food and drinking water. All experiments followed the Guidelines on Ethical Standards for Investigation of Experimental Pain in Animals [51] and the Mexican regulation (NOM-062-ZOO-1999). In addition, these experiments were approved by our local Ethics Committee (Protocol 455-09, Cinvestav, México City, México).

### Induction of spinal nerve ligation and measurement of tactile allodynia

Rats were prepared according to the method of [52]. Briefly, animals were anesthetized with a mixture of ketamine (50 mg/kg, i.p.) and xylazine (10 mg/kg, i.p.). After surgical preparation and exposure of the dorsal vertebral column, the left L5 and L6 spinal nerves were exposed and tightly ligated with 6-0 silk suture distal to the dorsal root ganglion. For sham-operated rats, the nerves were exposed but not ligated. Tactile allodynia was determined according to a previously reported method [53]. Fourteen days after surgery, rats were transferred to clear plastic, wire mesh-bottomed cages and allowed to acclimatize for 30–40 min. Von Frey filaments (Stoelting, Wood Dale, IL, USA) were used to determine the 50% paw withdrawal threshold using the up-down method [54]. Allodynia was considered to be present when paw withdrawal thresholds were less than 4 g.

### Spinal catheterization

Chronic catheterization of the spinal subarachnoid space was performed as described [55]. Seven or nine days after spinal nerve injury, rats were again anesthetized with a ketamine (45 mg/kg, i.p.)/xylazine (12 mg/kg, i.p.) mixture, placed in a stereotaxic head holder, and the atlantooccipital membrane exposed. The membrane was pierced, and a polyethylene catheter (PE-10, 7.5 cm length) was inserted intrathecally and advanced caudally to the level of the thoracolumbar junction. Rats were allowed to recover from surgery for 5 days in individualized cages before drugs administration and testing. For spinal drug administration, rats received an intrathecal (i.t.) injection of vehicle (10 μl) or increasing concentrations of the P2Y_6,11_ receptor antagonists (μM/10 μl). The antiallodynic effect was evaluated for the following 6 h in both conditions.

### Drugs

3-(2-Oxo-2-phenylethyl)-uridine-5'-diphosphate disodium salt (PSB0474), 4,4'-(carbonyl*bis*(imino-3,1-phenylene-carbonylimino-3,1-(4-methyl phenylene)carbonylimino))-*bis*(1,3-xylene-alpha,alpha'-diphosphonic acid tetrasodium salt (NF546), *N*,*N*"-1,4-butanediyl*bis*[*N*'-(3-isothiocyanatophenyl)thiourea (MRS2578) and 4,4'-(carbonyl*bis*(imino-3,1-(4-methyl-phenylene)carbonylimino))*bis*(naphthalene-2,6-disulfonic acid) tetrasodium salt (NF340) were purchased from Tocris Bioscience (Ellisville, MO). The purinergic drugs mentioned above were selected based on relevant receptor selectivity and efficacy. These included: (i) selective P2Y_6_ and P2Y_11_ receptors agonists PSB0474 [56] and NF546 [32], respectively, and (ii) selective P2Y_6_ and P2Y_11_ receptors antagonists MRS2578 [31] and NF340 [32], respectively. The doses used for P2Y_6,11_ receptor antagonists were chosen based in previous studies [28,32,57].

Minocycline hydrochloride (microglial inhibitor) and DL-fluorocitric acid barium salt (fluorocitrate, astrocytic inhibitor) were purchased from Sigma-Aldrich (St. Louis, MO). PSB0474, NF340 and NF546 were dissolved in 0.9% saline solution. MRS2578 and minocycline was dissolved in 20% DMSO. Fluorocitrate was dissolved initially in 2 M HCl (0.1 μmol/μL) and then diluted in 0.01 M PBS to attain a final concentration of 0.1 nmol/μL (pH 6.0). The vehicle control for fluorocitrate was 0.1% 2 M HCl in 0.01 M PBS (pH 6.0) [50].

### Western blot analysis

Western blot analysis was used to detect changes in the expression of P2Y_6,11_ receptors as well as Iba-1 and GFAP protein levels in the lumbar ipsilateral and contralateral dorsal spinal cord. For this purpose, naïve and ligated rats were sacrificed by decapitation. The lumbar region of the spinal cord (L4–L6) was sectioned to get the ipsilateral section of the dorsal spinal cord. Immediately, tissues were stored at −70°C. Tissues from individual animals were homogenized in ice-cold lysis buffer (in mM: 150 NaCl, 50 Tris–HCl, 5 EDTA), pH 7.4 for 30 min at 4°C. The protease inhibitors PMSF (1 mM), aprotinin (10 μg/mL), leupeptin (10 μg/mL), pepstatin A (10 μg/mL) and the surfactant 0.1% Triton X-100 (Sigma-Aldrich, St. Louis, MO) were added to the lysis buffer immediately prior to use. The homogenate was then centrifuged (Eppendorf, Hamburg) at 14,000 rpm for 10 min to remove cellular debris. The resultant supernatant was used to measure protein concentration by the Bradford’s method (Bio-Rad, Hercules, CA).

Fifty (for Iba-1 and GFAP) or one hundred (for P2Y_6_ and P2Y_11_ receptors) μg of proteins were resolved by denaturing by 10% or 12% (for Iba-1) SDS–polyacrylamide gel electrophoresis and transferred to PVDF membranes. Membranes were incubated in 5% non-fat milk in phosphate-buffered saline (PBS) at pH 7.4 (in mM: 137 NaCl, 2.7 KCl, 10 Na_2_HPO_4_ and 2 KH_2_PO_4_) with 0.1% Tween 20 for 1 h to block nonspecific proteins. After that, they were incubated overnight at 4°C in 5% non-fat dry milk/PBS containing rabbit anti-P2Y_6_ (BML-SA586-0050, 1:100, Enzo Life Sciences, Farmingdale, NY), rabbit anti-P2Y_11_ (AB9590, 1:500, Millipore, Billerica, MA), mouse anti-Iba-1 (MABN92, 1:500, Millipore, Bellerica, MA) or rabbit anti-GFAP (AB5804, 1:1000, Millipore, Bellerica, MA) antibodies. Membranes were incubated for 1 h at room temperature in 1% non-fat milk/PBS containing the horseradish peroxidase-conjugated secondary antibody (Donkey anti-rabbit, 711-035-152, 1:6000 or Donkey anti-mouse, 715-036-150, 1:5000; Both of Jackson immnunoresearch, West Grove, PA). Protein signal detection was achieved with the ECL chemiluminescence system (ECL plus, Amersham, UK). The next day, blots were stripped and incubated with a monoclonal antibody directed against β-actin (1:10000, MAB1501R, Millipore, Billerica, MA) which was used as an internal control to normalize P2Y_6_ and P2Y_11_ receptors and Iba-1 and GFAP protein expression levels. Scanning of the immunoblots was performed and the bands were quantified by densitometry using an image analysis program (LabWorks; UVP Inc., Upland, CA). In addition, control peptides for P2Y_6,11_ receptors were tested to determinate the bands specificity.

### Experimental design

In order to determine the role of spinal P2Y_6,11_ receptors in the neuropathic pain induced by spinal nerve ligation, neuropathic animals (14 days later) were intrathecally administered with vehicle or increasing doses of the selective P2Y_6_ (MRS2578, 10–100 μM) and P2Y_11_ (NF340, 0.3–30 μM) receptor antagonists.

As selective P2Y_6,11_ receptor antagonists produced antiallodynic effects, we next determined the expression of these receptors at the ipsilateral and contralateral dorsal spinal cord at 1, 3, 7 and 14 days after injury. Furthermore, to confirm the participation of the spinal P2Y_6,11_ receptors in the processing of neuropathic pain in rats, the expression of these receptors in absence and presence of the selective P2Y_6,11_ receptor antagonists was determined. For this, we administered the P2Y_6_ (100 μM) and P2Y_11_ (30 μM) receptor antagonists every 12 h for 3 days starting on day 12 after ligation. Rats were sacrificed on day 14 after nerve ligation.

In order to investigate the role of microglia and astrocytes in spinal nerve injury-induced tactile allodynia, rats were ligated and intrathecally canulated. On day 14, these animals received a daily intrathecal injection of vehicle, minocycline (100 μg/day) or fluorocitrate (1 nmol/day) for seven days. Tactile allodynia was determined 24 h after the first administration and then every other day until day 21. Rats were sacrificed on day 21 to determine the protein expression of Iba-1 and GFAP in the ipsilateral and contralateral dorsal spinal cord.

As our data showed the participation of microglia and astrocytes in neuropathic pain induced by spinal nerve ligation, we next investigated the role of the spinal purinergic P2Y_6,11_ receptors in the same conditions. To further confirm the participation of glial cells and spinal P2Y_6,11_ receptors, we next evaluated the effect of selective P2Y_6_ (PSB0474, 3–30 μM) and P2Y_11_ (NF546, 1–10 μM) receptor agonists in rats previously treated with minocycline or fluorocitrate for 7 days.

### Data analysis and statistics

All behavioral results are given as the mean ± S.E.M. for six animals per group. Curves were constructed by plotting the threshold for paw withdrawal as a function of time. An increase of 50% withdrawal threshold was considered as antiallodynia.

For protein expression, all results are given as the mean relative intensity ± S.E.M. for 3 animals per group.

One- or two-way analysis of variance, followed by the Student-Newman-Keuls was used to compare differences between groups. Differences were considered to reach statistical significance when *p* < 0.05.

## Abbreviations

ADP: Adenosine diphosphate; ATP: Adenosine triphosphate; BDNF: Brain derived neurotrophic factor; CCL-2: Chemokine ligand 2; CCL-3: Chemokine ligand 3; GFAP: Glial fibrillary acidic protein; Iba-1: Ionized calcium-binding adapter molecule-1; IL-1β: Interleukin-1β; IL-6: Interleukin-6; MRS2578: *N*,*N*"-1,4-Butanediyl*bis*[*N*'-(3-isothiocyanatophenyl)thiourea; NF340: 4,4'-(Carbonyl*bis*(imino-3,1-(4-methyl-phenylene)carbonylimino))*bis*(naphthalene-2,6-disulfonic acid) tetrasodium salt; NF546: 4,4'-(Carbonyl*bis*(imino-3,1-phenylene-carbonylimino-3,1-(4-methyl phenylene)carbonylimino))-*bis*(1,3-xylene-alpha,alpha'-diphosphonic acid tetrasodium salt; PBS: Phosphate-buffered saline; PSB0474: 3-(2-Oxo-2-phenylethyl)-uridine-5'-diphosphate disodium salt; TNFα: Tumor necrosis factor α; UDP: Uridine diphosphate.

## Competing interests

The authors declare that they have no competing interests.

## Authors’ contributions

PB-I carried out the molecular and pharmacological studies, performed the statistical analysis and drafted the manuscript. JBP-F carried out the molecular studies. CC-D participated in the pharmacological studies. MB-H participated in the molecular and pharmacological studies. HIR-G participated in the design and coordination of the study and helped to draft the manuscript. JM participated in the design, coordination and helped to draft to manuscript. VG-S conceived the idea, participated in the design and coordination of the study. He also helped to interpret the data and to draft the final manuscript. All authors read and approved the final manuscript.

## Authors’ information

PB-I, JBP-F, CC-D and MB-H are graduate students at the Department of Pharmacobiology in Cinvestav, South Campus. Mexico City, Mexico. HIR-G is a full professor at the Sección de Estudios de Posgrado e Investigación at the Escuela Superior de Medicina, Instituto Politécnico Nacional, Mexico City, Mexico. JM is a full professor at the Department of Pharmacobiology in Cinvestav, South Campus. Mexico City, Mexico. VG-S is a full professor at the Department of Pharmacobiology in Cinvestav, South Campus. Mexico City, Mexico.

## Supplementary Material

Additional file 1: Figure S1 Spinal nerve injury enhances the expression of P2Y_6,11_ receptors at 21 days after injury. Western blot analysis of the P2Y_6_ and P2Y_11_ receptors expression in the ipsilateral dorsal spinal cord obtained 21 days after nerve injury from naïve and spinal nerve injured rats. Data were normalized against β-actin and are expressed as the mean ± S.E.M. of 3 independent rats. *Significantly (*p* < 0.05) different from the naïve group, as determined by one-way analysis of variance followed by the Student-Newman-Keuls test. Click here for file

Additional file 2: Figure S2 Full blots of the P2Y_6_ and P2Y_11_ protein expression in the contralateral dorsal spinal cord. Western blot analysis of the P2Y_6_ (panel A) and P2Y_11_ (panel B) receptors expression in the contralateral dorsal spinal cord obtained from naïve (N) and spinal nerve injured rats (SNL). Data were normalized against β-actin and are expressed as the mean ± S.E.M. of 3 independent rats. Click here for file

Additional file 3: Figure S3 Repeated intrathecal minocycline or fluorocitrate reduces tactile allodynia: Effects of P2Y_6,11_ receptor agonists. Effect of the intrathecal treatment with minocycline (green symbols) or fluorocitrate (purple symbols) in spinal nerve injured rats (panels A-D). Time course of the allodynic effect of PSB0474 and NF546 in rats previously treated with repeated intrathecal minocycline (panels A and B) or fluorocitrate (panels C and D), respectively. Data are expressed as mean ± S.E.M. for 6 animals. *Significantly (*p* < 0.05) different from the vehicle (Veh) group, as determined by two-way analysis of variance followed by the Student-Newman-Keuls test. Click here for file
