# Supplementary figures and images for: Role of spinal P2Y6 and P2Y11 receptors in neuropathic pain in rats: possible involvement of glial cells
Source: Mol Pain. 2014 May 20;10:29. doi: 10.1186/1744-8069-10-29 (PMC4039548; doi:10.1186/1744-8069-10-29)

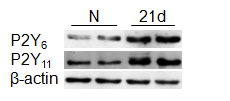


Figure S1. Barragán-Iglesias et al

Supplement: Additional file 1: Figure S1 — Spinal nerve injury enhances the expression of P2Y6,11 receptors at 21 days after injury. Western blot analysis of the P2Y6 and P2Y11 receptors expression in the ipsilateral dorsal spinal cord obtained 21 days after nerve injury from naïve and spinal nerve injured rats. Data were normalized against β-actin and are expressed as the mean ± S.E.M. of 3 independent rats. *Significantly (p < 0.05) different from the naïve group, as determined by one-way analysis of variance followed by the Student-Newman-Keuls test. [file 1744-8069-10-29-S1.docx]

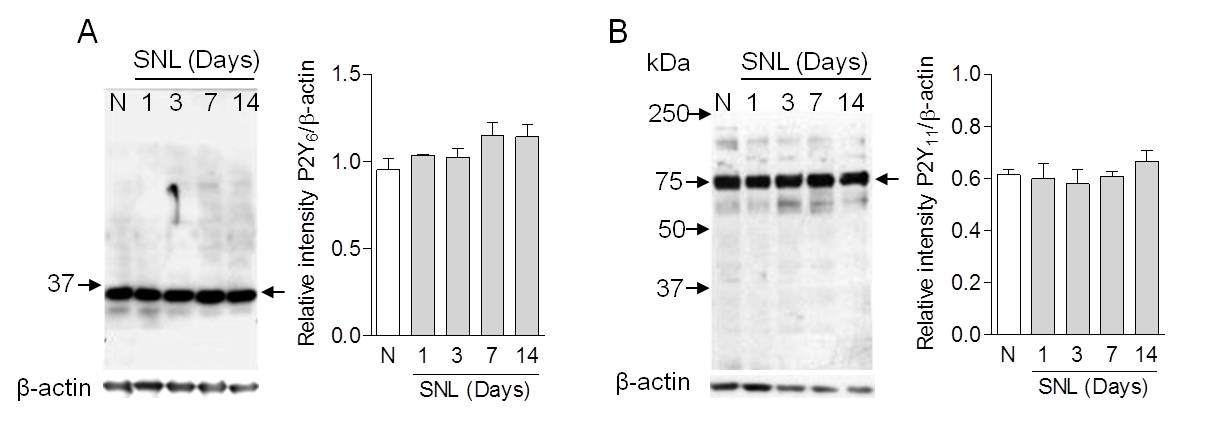


Figure S2. Barragán-Iglesias et al

Supplement: Additional file 2: Figure S2 — Full blots of the P2Y6 and P2Y11 protein expression in the contralateral dorsal spinal cord. Western blot analysis of the P2Y6 (panel A) and P2Y11 (panel B) receptors expression in the contralateral dorsal spinal cord obtained from naïve (N) and spinal nerve injured rats (SNL). Data were normalized against β-actin and are expressed as the mean ± S.E.M. of 3 independent rats. [file 1744-8069-10-29-S2.docx]
