# Supplementary material for: Role of spinal P2Y6 and P2Y11 receptors in neuropathic pain in rats: possible involvement of glial cells
Source: Mol Pain. 2014 May 20;10:29. doi: 10.1186/1744-8069-10-29 (PMC4039548; doi:10.1186/1744-8069-10-29)
Supplement: Additional file 3: Figure S3 — Repeated intrathecal minocycline or fluorocitrate reduces tactile allodynia: Effects of P2Y6,11 receptor agonists. Effect of the intrathecal treatment with minocycline (green symbols) or fluorocitrate (purple symbols) in spinal nerve injured rats (panels A-D). Time course of the allodynic effect of PSB0474 and NF546 in rats previously treated with repeated intrathecal minocycline (panels A and B) or fluorocitrate (panels C and D), respectively. Data are expressed as mean ± S.E.M. for 6 animals. *Significantly (p < 0.05) different from the vehicle (Veh) group, as determined by two-way analysis of variance followed by the Student-Newman-Keuls test. [file 1744-8069-10-29-S3.docx]

**C**

**D**

Figure S3. Barragán-Iglesias et al

**C**
